# Defining the gut microbiota in individuals with periodontal diseases: an exploratory study

**DOI:** 10.1080/20002297.2018.1487741

**Published:** 2018-07-03

**Authors:** Talita Gomes Baeta Lourenςo, Sarah J. Spencer, Eric John Alm, Ana Paula Vieira Colombo

**Affiliations:** a Department of Medical Microbiology, Institute of Microbiology, Federal University of Rio de Janeiro, Rio de Janeiro, Brazil; b Computational and Systems Biology, Massachusetts Institute of Technology, Cambridge, MA, USA; c Department of Biological Engineering, Massachusetts Institute of Technology, Cambridge, MA, USA; d Center for Microbiome Informatics and Therapeutics, Massachusetts Institute of Technology, Cambridge, MA, USA

**Keywords:** Gut microbiome, periodontal diseases, oral microorganisms, microbial metagenome, human microbiome

## Abstract

**Background**: This exploratory study aimed to characterize the gut microbiome of individuals with different periodontal conditions, and correlate it with periodontal inflammation and tissue destruction.

**Methods**: Stool samples were obtained from individuals presenting periodontal health (PH = 7), gingivitis (*G* = 14) and chronic periodontitis (CP = 23). The intestinal microbiome composition was determined by Illumina MiSeq sequencing.

**Results**: A lower alpha-diversity in the gut microbiome of individuals with CP was observed, although no significant difference among groups was found (*p* > 0.01). *Firmicutes, Proteobacteria, Verrucomicrobia* and *Euryarchaeota* were increased, whereas *Bacteroidetes* were decreased in abundance in patients with periodontitis compared to PH. *Prevotella* (genus), *Comamonadaceae* (family) and *Lactobacillales* (order) were detected in higher numbers in G, while *Bacteroidales* (order) was predominant in PH (*p* < 0.01). Significant correlations (rho = 0.337–0.468, *p* < 0.01) were found between OTUs representative of periodontal pathogens and attachment loss. *Mogibacteriaceae, Ruminococcaceae* and *Prevotella* were able to discriminate individuals with periodontal diseases from PH (overall accuracy = 84%). Oral taxa were detected in high numbers in all stool samples.

**Conclusions**: Individuals with periodontal diseases present a less diverse gut microbiome consistent with other systemic inflammatory diseases. High numbers of oral taxa related to periodontal destruction and inflammation were detected in the gut microbiome of individuals regardless of periodontal status.

## Introduction

Disturbance on human microbiota colonizing the various body sites has been implicated in a wide range of microbiome-related inflammatory diseases [1–5]. Among those, periodontal diseases are complex polymicrobial inflammatory diseases associated with dysbiosis of the dental biofilm that induces a long-lasting chronic inflammation of the periodontal supporting tissues, leading to alveolar bone destruction, and eventual tooth loss [6]. Over the years, strong evidence has accumulated to indicate that the pathogenic microbiota and the chronic inflammation established in periodontitis contribute to the onset and/or progression of several systemic inflammatory diseases such as cardiovascular diseases [7,8], diabetes [9], obesity [10], metabolic syndrome [11], respiratory disease [12], cancer [13], chronic kidney disease (CKD) [14] and rheumatoid arthritis (RA) [15]. Most research on the periodontitis-systemic disease relationship, however, has not determined causality, and the link between these diseases are bi-directional associations [16].

There are several biologically plausible mechanisms to support these associations. The direct or indirect effects of circulating bacteria, inflammatory mediators and/or immune complexes from infected/inflamed periodontal tissues on other body sites are some of the main mechanisms that contribute to systemic inflammation [17]. Oral bacteria can enter the circulation and cause bacteraemia by actively crossing the periodontal epithelium [18–20], or by being inoculated through mechanical procedures, including periodontal debridement, flossing and brushing [21,22]. Periodontal pathogens, such as *Aggregatibacter actinomycetemcomitans, Treponema denticola* and *Porphyromonas gingivalis* are also capable of invading endothelial cells [23–27], and they have been detected in atherosclerotic plaques, heart valves, aortic aneurysms, carotid and coronary vessels [28–33]. Studies in a variety of animal models have demonstrated that recurrent bacteraemia or oral administration with *P. gingivalis* can enhance atherogenesis [34,35]. Of interest, *P. gingivalis* is so far the only bacterium capable of causing enzymatic citrullination of peptides with subsequent development of anti-citrullinated peptide auto-antibodies, a major etiopathologic event in RA [36]. Bacterial by-products, particularly LPS from the predominant Gram-negative periodontal biofilm may also contribute to systemic inflammation. Wahaidi et al. [37] showed a significant increase in the levels of systemic endotoxin and in hyperactivity of circulating neutrophils following 21 days of dental plaque accumulation (experimental gingivitis). After treatment of gingivitis, a reduction of endotoxemia to baseline levels was observed.

Alternatively, data have suggested that the inflammatory response to periodontal bacteria at the inflamed periodontal tissues represents a source of persistent chronic systemic inflammation [38]. Pro-inflammatory mediators and biomarkers are significantly more elevated in serum and gingival crevicular fluid of individuals with periodontitis compared to periodontally healthy individuals [38–41]. In addition, periodontal treatment generally lowers most of these mediators [41,42].

Another possible mechanism linking periodontitis and inflammatory systemic diseases would be through a disturbance of the gut microbiome by a long-term, orally ingested high dose of periodontopathic microorganisms. Based on this hypothesis, individuals with chronic periodontal diseases would eventually establish a disturbed gut microbiome commonly seen in individuals affected by systemic inflammatory diseases. In fact, the novel pathogenesis model of periodontitis (the ‘keystone-pathogen hypothesis’) proposes that periodontal pathogens can orchestrate inflammatory periodontal disease by remodelling a symbiotic periodontal microbiota into a dysbiotic one, as demonstrated in animal model studies [43,44]. However, no clinical studies in humans have evaluated the ability of periodontal pathogens to cause a dysbiosis in the gut microbiome. So far, only two studies in mice have shown that oral administration of *P. gingivalis* induces increased local and systemic inflammation, and significant changes in the gut microbiota composition [45,46]. Furthermore, the gut microbial profile of systemically healthy individuals with periodontal diseases has not been explored.

Considering that periodontitis patients, commonly colonized by higher levels of periodontal pathogens in the sub-gingival biofilm, may present a unique gut microbiota, we aimed to determine and compare the composition of the gut microbiome of individuals with gingivitis and chronic periodontitis to periodontally healthy controls in a parallel observational case-control study, using high-throughput sequencing of the 16S rRNA gene, and to correlate this microbiome with parameters of periodontal inflammation and tissue destruction.

## Materials and methods

### Study population

The population of this study was recruited from the Division of Graduate Periodontics of the School of Dentistry at the Federal University of Rio de Janeiro (UFRJ), between January 2015 and March 2016. Participants were individually informed about the nature of the study, its risks and benefits, and signed informed consent forms. To be enrolled, patients had to be ≥ 18 years of age, have ≥ 18 teeth, and be in good general health. Exclusion criteria included history of periodontal treatment and use of topical or systemic antimicrobials in the last 6 months; and use of anti-inflammatory drugs in the last 3 months previous to the initial examination; need for chemoprophylaxis; presence of diabetes, immune-deficiencies, chronic gastrointestinal diseases or abnormal gastrointestinal symptoms; history of metabolic disease; Body Mass Index (BMI) ≥30; pregnancy and nursing. This study was conducted according to the principles outlined in the Declaration of Helsinki of 1975 on experimentation involving human subjects, revised in 2000. The study protocol was approved by the Human Research Ethics Committee of the Hospital of the Federal University of Rio de Janeiro (UFRJ), Brazil (approval #685.070).

### Clinical examination

At the first visit, individuals answered anamnesis questionnaires and data on age, gender, race, smoking and lifestyle (eating habits, physical activity practice and use of alcoholic beverages). Clinical examinations were performed by calibrated periodontists and included probing depth (PD) and clinical attachment level (CAL), presence of bleeding on probing (BOP), gingival bleeding (GI), visible supragingival plaque (PL), calculus (CA) and suppuration (SUP). Individuals were diagnosed as having periodontal health (PH), gingivitis (G), and chronic periodontitis (CP) according to Silva-Boghossian et al. [47]. Briefly, periodontal health was defined as ≤ 10% of sites with BOP and/or GI, no PD or CAL > 3 mm, although PD or CAL = 4 mm in up to 5% of sites without BOP was allowed. Gingivitis was defined as > 10% of sites with BOP and/or GI, no PD or CAL> 3 mm, although PD or CAL = 4 mm in up to 5% of sites without BOP was allowed. Chronic periodontitis was defined as > 10% of teeth with PD and CAL ≥ 5 mm with BOP.

### Collection and processing of faecal samples

Patients were instructed to return within one week after clinical examination with a fresh sample of faeces preserved into a sterile recipient previously provided. The samples were immediately processed for extraction and purification of genomic DNA (gDNA). Briefly, 1g of faeces was diluted in 10 mL of lysis buffer (0.5M Tris-HCl, 20 mM EDTA, 10 mM NaCl, 0.1% SDS and pH 9.0), vortexed for 5 min and homogenized for 10 min. A further dilution (1:2) was made in lysis buffer. The samples were homogenized again for 5 min and centrifuged at 12,000*x g* for 10 min [48]. The supernatant was transferred to a new tube, centrifuged and re-suspended in 150 μl of TE buffer. The samples were then incubated with 10 μl of lysozyme (20 mg/mL) overnight at 37°C prior to initiating extraction using a commercial kit, according to the manufacturer’s instructions (MasterPure DNA Purification Kit, Epicentre, Madison, WI). Measurements and purity of DNA samples were evaluated by spectrophotometry using a Nano Drop Lite™ (ThermoFisher Scientific, São Paulo, SP). Random samples were also evaluated by agarose gel electrophoresis (1.5%). Samples were stored at −80°C.

### Illumina library preparation for 16S rRNA gene amplicons

Samples were randomly arrayed onto multi-well plates for library preparation. Both positive and negative controls were included on the plates for each amplification reaction. Our library preparation protocol consisted of two main amplification steps, one to amplify and tag 16S rRNA variable regions, and a second to add final Illumina adapters. Prior to the first amplification, we completed duplicate qPCRs with 1:20 and 1:200 dilutions of gDNA to determine relative concentrations and normalize the input gDNA. These reactions targeted the 16S rRNA gene V4 variable region with primers PE16S_V4_U515_F and PE16S_V4_E786R (Table S1). The reactions included 0.5X SYBR Green I nucleic acid gel stain (Sigma-Aldrich, St. Louis, MO), 280 nM of each primer, and the standard Phusion High-Fidelity PCR Kit (New England BioLabs, Ipswich, MA) reagents according to the manufacturer’s instructions. Cycling conditions for qPCR then included 98°C for 30 sec; 30 cycles of 98°C for 30 sec, 52°C for 30 sec, 72°C for 30 sec; and 4°C hold. The threshold Ct values were used to quantify relative concentrations of samples. These quantifications allowed us to prepare normalized dilutions of all the samples for the first step PCR. We completed the first step PCR under the same conditions described for the qPCR earlier, minus the 0.5X SYBR Green I. We set the number of cycles based on Ct calculations and dilutions described. The reactions were run in quadruplicate for each sample and then pooled after thermal cycling. Each pooled reaction was purified using AgencourtAMPure XP Beads according to the manufacturer’s instructions (Beckman Coulter, Brea, CA), and 1/4 of the final elution volume was used as input into the final library amplification. The final library amplification was conducted to add Illumina adapter sequences and sample-specific barcodes to either end of the constructs. We used the Phusion High-Fidelity PCR Kit according to manufacturer’s instructions, and added 420 nM each of indexing primers PE-III-PCR-F and PE-IV-PCR-R (Table S1). These primers were added row- and column-wise respectively to array 96 barcodes from eight forward and 12 reverse primers. Thermal cycling conditions included 98°C for 30 sec; seven cycles of 98°C for 30 sec, 83°C for 30 sec, 72°C for 30 sec; and 4°C hold. These were single reactions for each sample that proceeded immediately into AgencourtAMPure XP Bead purification according to the manufacturer’s protocol. Samples were quantified with SYBR Green I and a standard curve, then pooled in equimolar ratios for 2 × 250 bp paired-end sequencing on an Illumina MiSeq. All Illumina sequence data from this study were submitted to Sequence Read Archive (SRA) under BioProject accession number SRP115612.

### Sequencing data processing

Raw reads were quality filtered and clustered into operational taxonomic units (OTUs) with the QIIME pipeline version 1.9.1 [49], using default parameters unless otherwise noted. After quality filtering (split_libraries_fastq.py – barcode_type16, – min_per_read_length_fraction 0.40, -q 20, – max_barcode_errors 0, – max_bad_run_length 0, – phred_offset 33), reads were clustered at the 97% similarity level, classified against the 16S rRNA GreenGenes database, as well as the Human Oral Microbiome Database (HOMD) RefSeq version 14.5, for oral taxa analysis, and aligned in order to build phylogenetic trees. We ran the QIIME commands pick_otus.py, pick_rep_set.py (-m most_abundant), and make_otu_table.py to produce the OTU table. The uclust classifier was used to assign taxonomy with default parameters. The frequency of detection of each OTU was computed for each sample. Also, the number of reads assigned to each OTU was counted and normalized to relative abundance. A rarefaction stage was performed, standardizing the samples for a total of 10,000 sequences per sample. The alpha diversity was calculated using the Shannon indices, Faith’s phylogenetic diversity [50], OTU richness, and Chao1 index, and compared between groups using nonparametric two sample *t*-tests, using the default Monte Carlo permutations. The beta diversity was performed using the Weighted Distance Matrix Analysis (UniFrac), calculated by the difference in probability of mass of OTUs of each community for each branch [51] and Principal Coordinates Analysis (PCoA) plots to evaluate the degree of variation among the samples. The ANOSIM test was used to compare the ranged distances of beta diversity between groups, and to calculate the correlation coefficient and *p* value by permutation. The generated OTU tables combined with the clinical data were used as input for follow-up analysis.

### Statistical analyses

Statistical analyses were performed using the SPSS programme (Statistical Package for the Social Sciences 21.0, IBM Brazil, São Paulo, Brazil). For demographic data, frequency and means were computed for each patient and group. Periodontal clinical parameters were averaged for each patient and then across groups. Comparisons among groups were evaluated by Chi-square (for categorical data), Mann-Whitney (for pairs of groups) and Kruskal-Wallis tests. The relative abundance at the phylum and genera taxonomic levels were calculated for each patient and averaged within each clinical group. Comparisons among groups were evaluated by the Mann-Whitney and Kruskal-Wallis tests. From the raw OTU tables generated, the assigned OTUs detected at numbers ≥ 50 in all 44 samples were computed as mean counts of reads within each group. Associations between gut OTUs, periodontal inflammation (BOP and GI) and tissue destruction (PD and CAL) were evaluated by correlation analysis of Spearman. Random forest analysis using the out-of-bag method of prediction error was carried out to classify the clinical status of individuals based on the number of reads of different OTUs. The mean decrease in accuracy was assessed for each OTU to determine the variables of importance for prediction by removing the association between that variable and the target (clinical status). This was achieved by randomly permuting the values of the variable and measuring the resulting increase in error. For all analyses, significance level was set at 1%.

## Results

### Clinical features of the study population

A total of 82 patients were selected according to the criteria of inclusion. Of those, 44 were included into the analysis. These patients were diagnosed as having periodontal health (PH, *n* = 7), gingivitis (G, *n* = 14) or chronic periodontitis (CP, *n* = 23). Demographic, lifestyle, diet and clinical data of the study population are presented in Table S2. Regarding lifestyle and diet, gender, smoking status, and race, no significant differences among groups were found. However, patients in the CP group presented significantly greater mean BMI compared to the PH and G patients (Mann-Whitney test, p < 0.01). Nevertheless, no differences among groups were seen for diet (data not shown). Individuals in the CP group were significantly older than individuals with G (Mann-Whitney test, *p* < 0.01). Periodontitis patients presented significantly more periodontal destruction, calculus and missing teeth than PH and G patients (Kruskal-Wallis test, *p* < 0.01). Regarding inflammation and supragingival plaque, G and CP individuals presented more sites with BOP, GI and PL than controls, and no differences were seen between both diseased groups (Mann-Whitney test, *p* < 0.01).

### Gut microbiome samples of diseased cohorts showed low diversity

Sequencing of the 253 bp segment corresponding to the V4 region of the gene encoding the 16S rRNA from the stool samples of 49 patients generated 2,508,767 sequences. After screening and rarefaction, samples from 44 patients (PH, *n* = 7, G, *n* = 14, CP, *n* = 23) were included in the analyses. A total of 957,061 sequences were clustered into 1367 identifiable OTUs, and a total of 1093 sequences were classified as unassigned. The mean number of reads per sample assigned to OTUs was 21,751, ranging from 2547 to 101,648.

**Figure 1.**
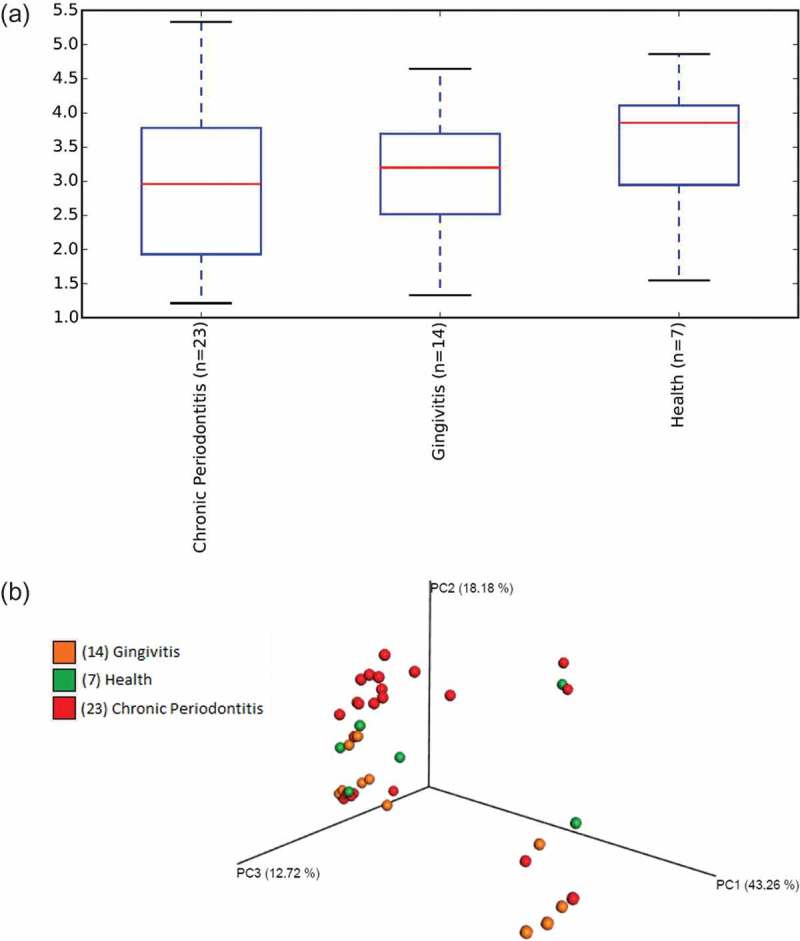
High diversity of the gut microbiome among individuals with different periodontal conditions. **(a)** Alpha diversity based on relative abundance, using Shannon indices, calculated for each clinical group (*p* > 0.05, Student *t* test). **(b)** Beta diversity for comparison of microbial community composition among clinical groups (Periodontal Health, Gingivitis, Chronic Periodontitis). Weighted UniFrac analysis was used to generate distances among different samples, and plots were generated by using principal coordinate analysis (PCoA). The percentage of variation explained by each PC is indicated on the axes. No statistical differences among groups were observed (*R* = −0.0388, *p* = 0.758, ANOSIM test).

Different values of phylogenetic diversity among groups can be observed, with a decrease in diversity from a healthy periodontal condition (3.48 ± 1.01) to periodontitis (2.95 ± 1.15); however, no significant differences among groups were observed (Figure 1(a)). Beta-diversity analysis compared bacterial communities based on their compositional structures and resulted in a PCoA (distance matrix), showing the spatial separation of the samples, with different colours indicating the clinical groups (Figure 1(b)). Seventy-four per cent of the total variance among the individual samples were explained by the first three principal components (PCs). The PC1 axis was the one with the greatest contribution, accounting for 43.3% of the variation found in the microbiota. PC2 and PC3 explained, respectively, 18.18% and 12.72% of the inter-sample variations (Figure 1(b)). It was not possible to clearly distinguish clinical status by microbial communities (*R* = −0.0388, *p* = 0.758, ANOSIM test).

### Increasing firmicutes, proteobacteria, verrucomicrobia and euryarchaeota phyla in subjects with periodontitis

**Figure 2.**
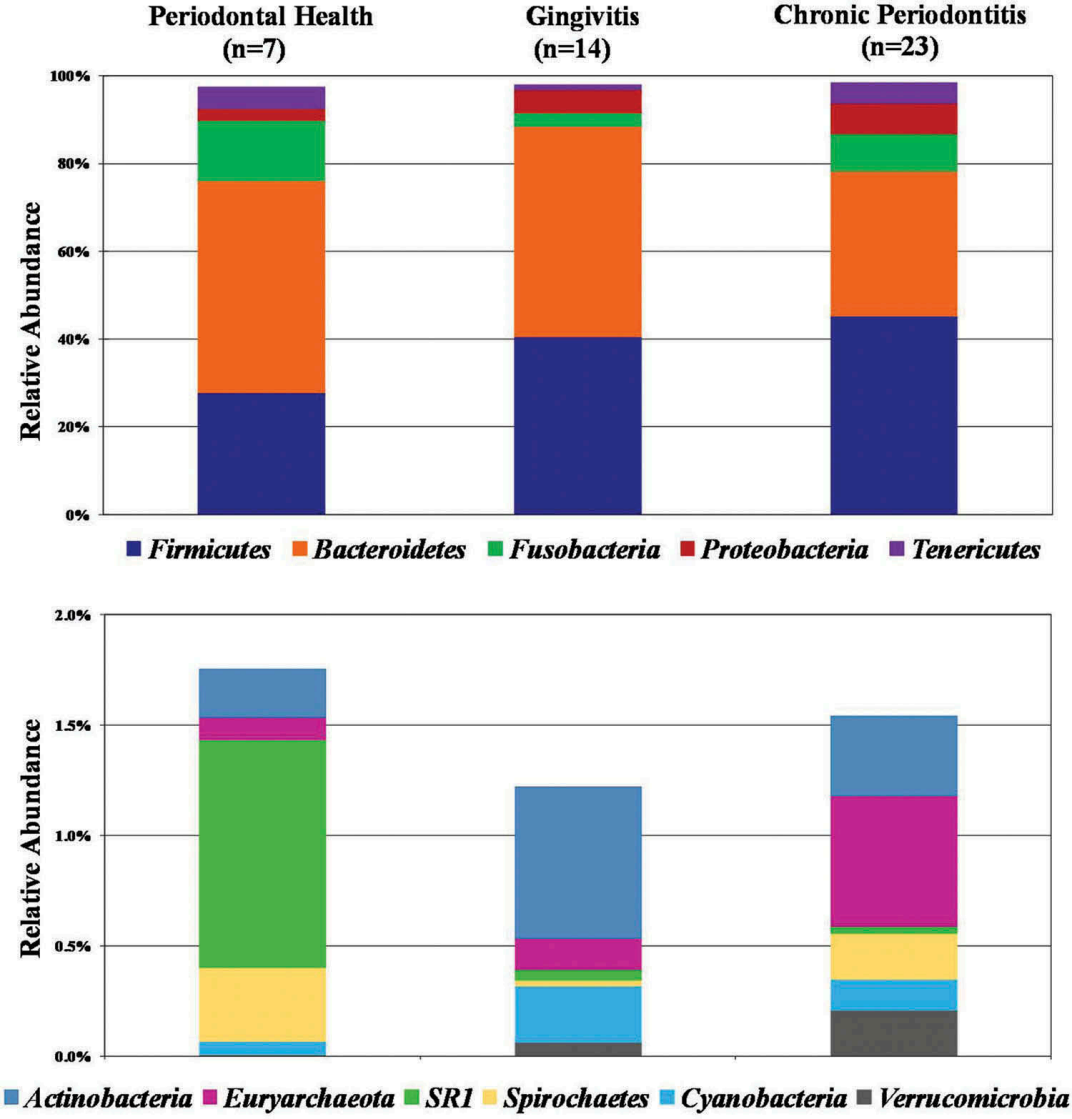
Proportional taxonomic assignments at the phylum level in stool samples from individuals with different periodontal status. Only phyla detected at mean relative abundance ≥ 0.1% are presented. Upper panel correspond to phyla detected in higher mean relative abundance, and lower panel represent the phyla in low abundance. No significant differences among groups were observed (Mann-Whitney, Kruskal-Wallis test, *p* > 0.01).

Figure 2 and Figure S1 show the relative abundance of bacterial taxa at the phylum level in patients from all clinical groups. Fifteen different phyla were identified, with a predominance of *Firmicutes* (40.9%) and *Bacteroidetes* (40.5%), followed by *Fusobacteria* (7.6%), *Proteobacteria* (5.8%) and *Tenericutes* (3.6%). Phyla detected in low abundance included *Actinobacteria* (0.44%), *Euryarchaeota* (0.37%), SR1 (0.19%), *Spirochaetes* (0.17%), *Cyanobacteria* (0.16%), *Verrucomicrobia* (0.13%), *Lentisphaerae* (0.07%), *Synergistetes* (0.01%), *Elusimicrobia* (0.005%) and GN02 (0.0006%). The phyla *Firmicutes, Proteobacteria, Verrucomicrobia* and *Euryarchaeota* showed a tendency to increase in abundance in the diseased groups compared to PH, whereas *Bacteroidetes* were decreased in abundance in individuals with periodontitis. However, these differences in abundance between groups were not significant (Mann-Whitney, Kruskal-Wallis tests, *p* > 0.01). Even at this high taxonomic level, a great inter-individual variability in the proportions of these phyla can be seen in all groups (Figure S1).

### Genera related to periodontal pathogens were abundant in all clinical groups

**Figure 3.**
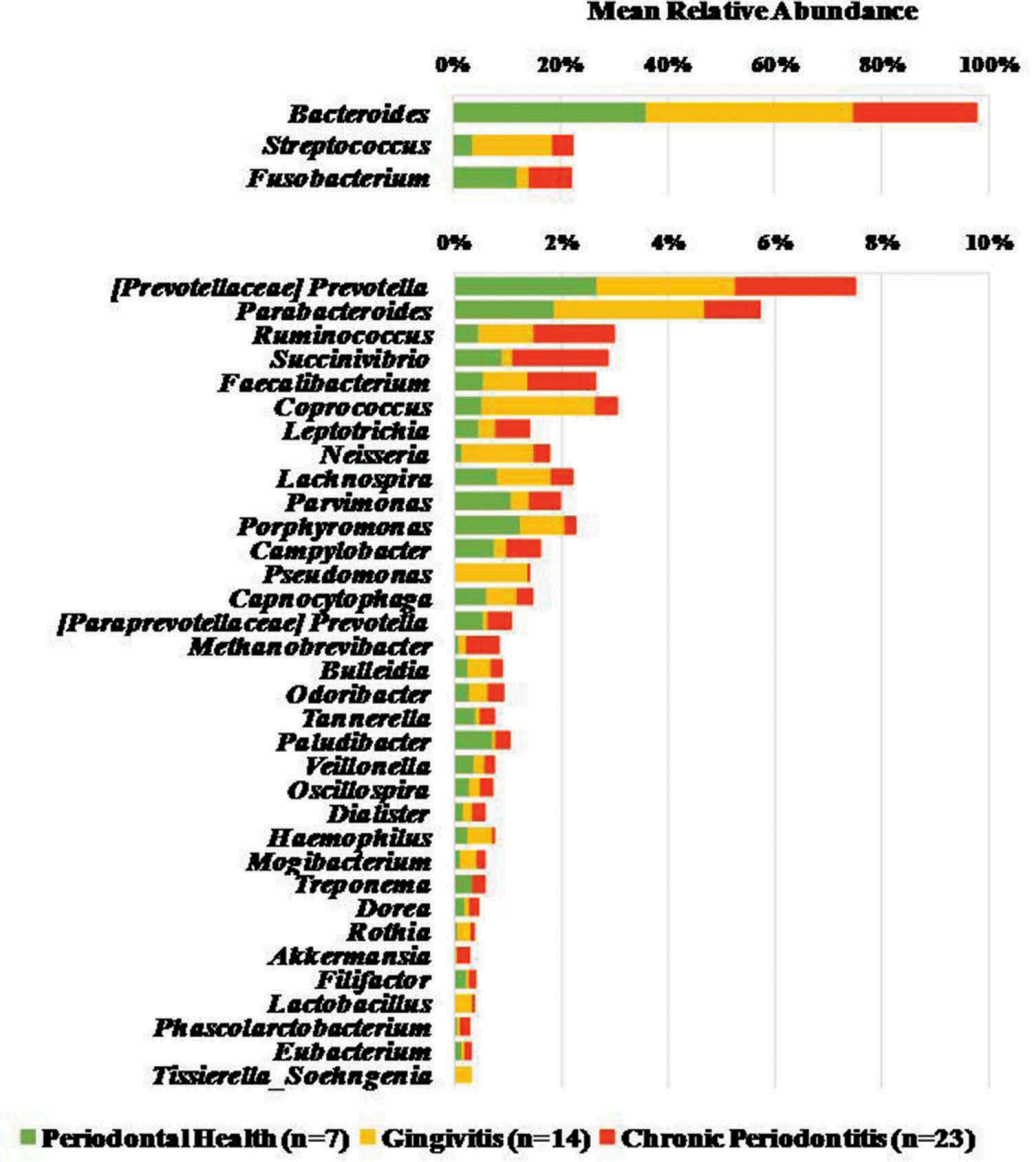
Proportional taxonomic assignments at the genus level in stool samples from individuals with different periodontal status. Genera detected at mean relative abundance ≥ 0.1% are presented. No significant differences among groups were observed (Mann-Whitney and Kruskal-Wallis tests, p > 0.01).

We also tested for significant differences between patient groups at finer taxonomic resolution. In particular, a total of 127 genera were detected in at least one sample. Genera detected at mean relative abundance ≥ 0.1% in all samples are presented in Figure 3. The most abundant genera were *Bacteroides* (29.4%), followed by *Streptococcus* (7.7%), *Fusobacterium* (6.7%) and *[Prevotellaceae] Prevotella* (2.8%). Although these genera accounted for most of the sequences obtained from all samples, variability in abundance among individuals within the same clinical groups could also be observed (Figure S2). Several genera related to putative periodontal pathogens such as *Fusobacterium, Prevotella, Parvimonas, Porphyromonas, Tannerella, Dialister, Filifactor, Treponema* and *Eubacterium* were detected in stool samples of all groups. No significant differences in abundance of these predominant genera were observed among groups (Mann-Whitney, Kruskal-Wallis tests, *p* > 0.01).

The mean number of reads for each OTU was also calculated and compared among clinical groups (Tables S3). Only assigned OTUs detected at 50 or more reads in all samples were included in these analyses. OTUs assigned to the same genus, family or order were grouped as representative OTUs, and their numbers averaged within each clinical group. OTU classifications present in high mean number of reads included *Clostridiales* (order), *Bacteroides* (genus), *[Mollicullites] RF39* (order), *Rikenellaceae* (family), *Fusobacterium* (g), *Streptococcus* (g), *Bacteroides uniformis* and *Ruminococcaceae* (f). Approximately 46% of the assigned OTUs were more abundant in diseased individuals (particularly in patients with gingivitis), while few classified OTUs were predominant in samples from PH individuals, including known oral pathogens such as *Porphyromonas endodontalis* and *Prevotella tannerae*. The numbers of OTUs assigned to the genus *Prevotella*, the family *Comamonadaceae*, and the order *Lactobacillales* were significantly more abundant in gingivitis patients than PH and CP individuals, whereas *Bacteroidales* (o) was detected in higher counts in PH (Kruskal-Wallis, Chi-square tests, *p* < 0.01).

### Prediction of periodontal disease determined by mogibacteriaceae, prevotella and ruminococcaceae in stool samples

We constructed random forest classifiers to attempt prediction of oral disease state based on the gut microbiome composition. Discrimination of PH and periodontal disease (G and CP) groups based on the number of OTUs has shown that all 37 diseased individuals were correctly classified, as demonstrated by the confusion matrix in Figure S3 (Overall prediction accuracy = 0.841). OTUs which improved prediction of periodontal disease relative to PH were determined in decreasing order of accuracy (Figure S3b). The families *Mogibacteriaceae* and *Ruminococcaceae*, and the genus *Prevotella* contributed most to model accuracy in classification of diseased patients. In contrast, none of the PH patients were predicted to be healthy, likely due to healthy variability in steady states (Figure S3a).

### Specific gut phylotypes correlate with periodontal inflammation and attachment loss

Regardless of the periodontal status, associations between mean number of OTU reads and periodontal inflammation and periodontal attachment loss were examined (Table 1). A subset of OTUs showed significant correlations (rho = 0.337–0.468, *p* < 0.01) with bleeding and periodontal tissue destruction. Of interest, genera and species associated with periodontal diseases showed significant moderate correlations with PD and CAL, including *Selenomonas noxia, Leptotrichia, Tannerella* and *Campylobacter* (*p* < 0.01).

**Table 1.** Correlation analysis between parameters of inflammation, attachment loss and OTUs detected in faeces samples from 44 individuals with different periodontal status.

OTUs	GI	BOP	PD	CAL
Christensenellaceae (f)		0.468*		0.468
Ruminococcus (g)		0.433		
Clostridium (g)	0.417			
Selenomonas noxia			0.415	
Oribacterium (g)				0.386
Leptotrichia (g)				0.382
Campylobacter (g)			0.370	
Clostridiales (o)	0.358			
Succinivibrio (g)	0.350			
Eubacterium biforme			0.340	
Tannerella (g)			0.337	

GI: gingival bleeding. BOP: bleeding on probing. PD: probing depth. CAL: clinical attachment level.(o): order taxonomic level; (f): family taxonomic level; (g): genus taxonomic level. *Refers to the Spearman coefficient (rho). All correlations presented were significant at *p* < 0.01.

### High oral taxa counts in the gut microbiome of individuals with gingivitis

OTUs representing traditionally oral species were frequently detected in stool samples across the entire patient population. Over 100 species/phylotypes representative of oral microorganisms were identified. OTUs of oral organisms within species- or genus-level taxonomic groups, detected at total numbers ≥ 100 reads in all samples were selected and their numbers averaged within groups. Figure 4 presents the mean number of reads for oral OTUs. The top 10 organisms detected in high mean reads were *Bacteroides heparinolyticus, Alloprevotella rava, Fusobacterium* spp., *Streptococcus australis, Tannerella* spp., *Lachnospiraceae* [G-2] spp., *Oribacterium* sp. OT102, *Prevotella* spp., *Prevotella maculosa* and *Neisseria* spp. Significant differences in oral taxa counts among groups were observed for *S. australis, Prevotella* spp., *Rothia eria, Granulicatella adiacens, Oribacterium asaccharolyticum* and *Porphyromonas* sp. OT930 (present in high mean reads in G compared to the other groups), and *Peptostreptococcaceae* [XI][G-6] which was predominant in PH individuals (Kruskal-Wallis, Mann-Whitney tests, *p* < 0.01). Other oral species, including several oral pathogens (*Prevotella maculosa, Prevotella veroralis, Prevotella denticola, Slackia exigua, Campylobacter curvus, Porphyromonas* sp. OT930 and OT279, *Dialister invisus, Peptostreptococcus stomatis, Porphyromonas endodontalis, Alloprevotella tannerae, Prevotella oulorum, Treponema maltophilum, Campylobacter rectus, Filifactor alocis* and *Parvimonas micra*) were detected across samples regardless of periodontal status (Figure 4).

**Figure 4.**
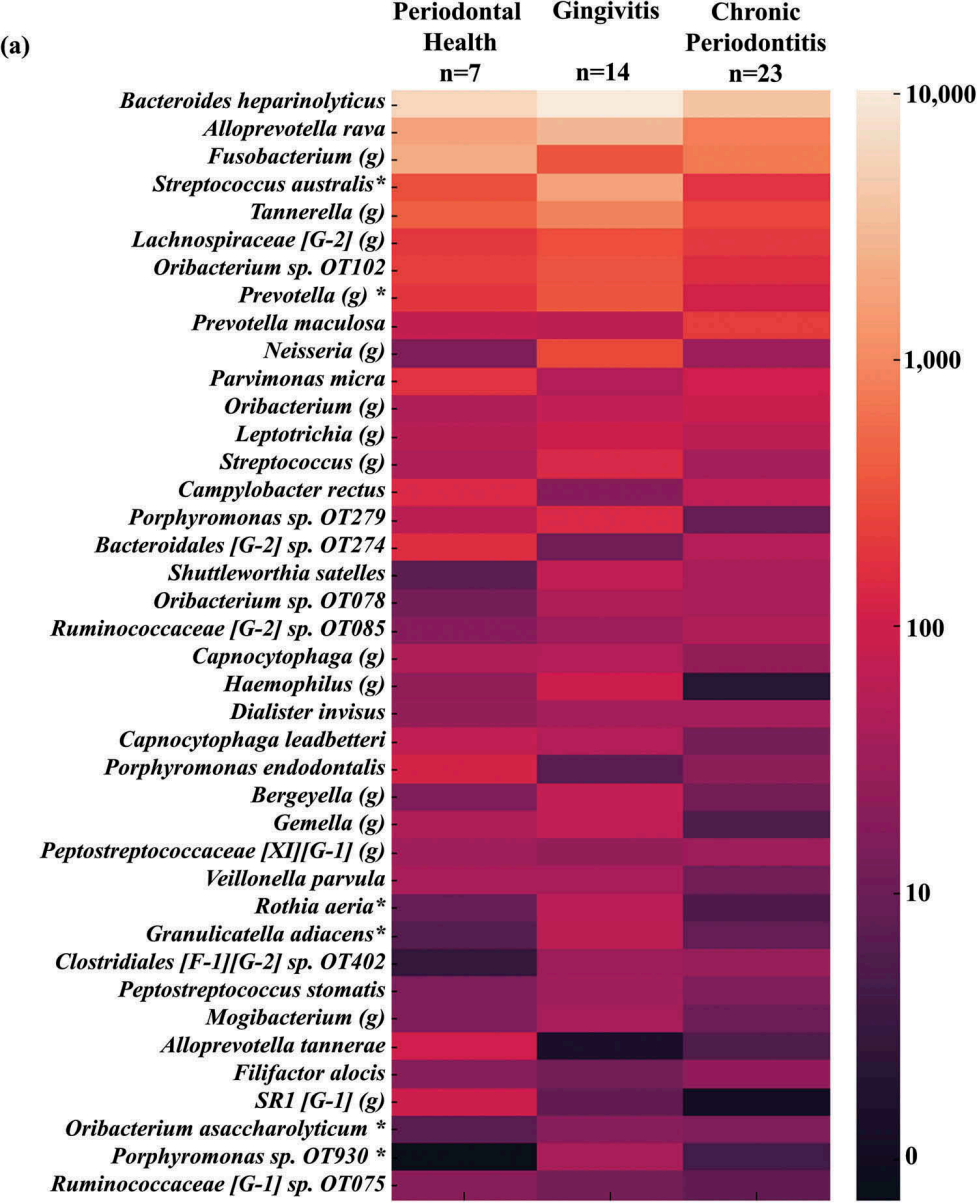
Oral taxa detected in the gut microbiota. A heat map of OTUs (mean number of reads) with > 97% identity to oral taxa, which were detected in stool samples from individuals with different periodontal status. **(a)** Oral taxa detected at mean numbers ≥ 10; **(b)** Oral taxa detected at lower mean numbers (< 10). *Significant differences among groups were observed for *S. australis, Prevotella* spp., *Rothia aeria, Granulicatella adiacens, Oribacterium asaccharolyticum, Porphyromonas* sp. OT930, and *Peptostreptococcaceae* (Kruskal-Wallis, Mann-Whitney tests, *p* < 0.01). OT: oral taxon. (g) genus.

**Figure 4.**
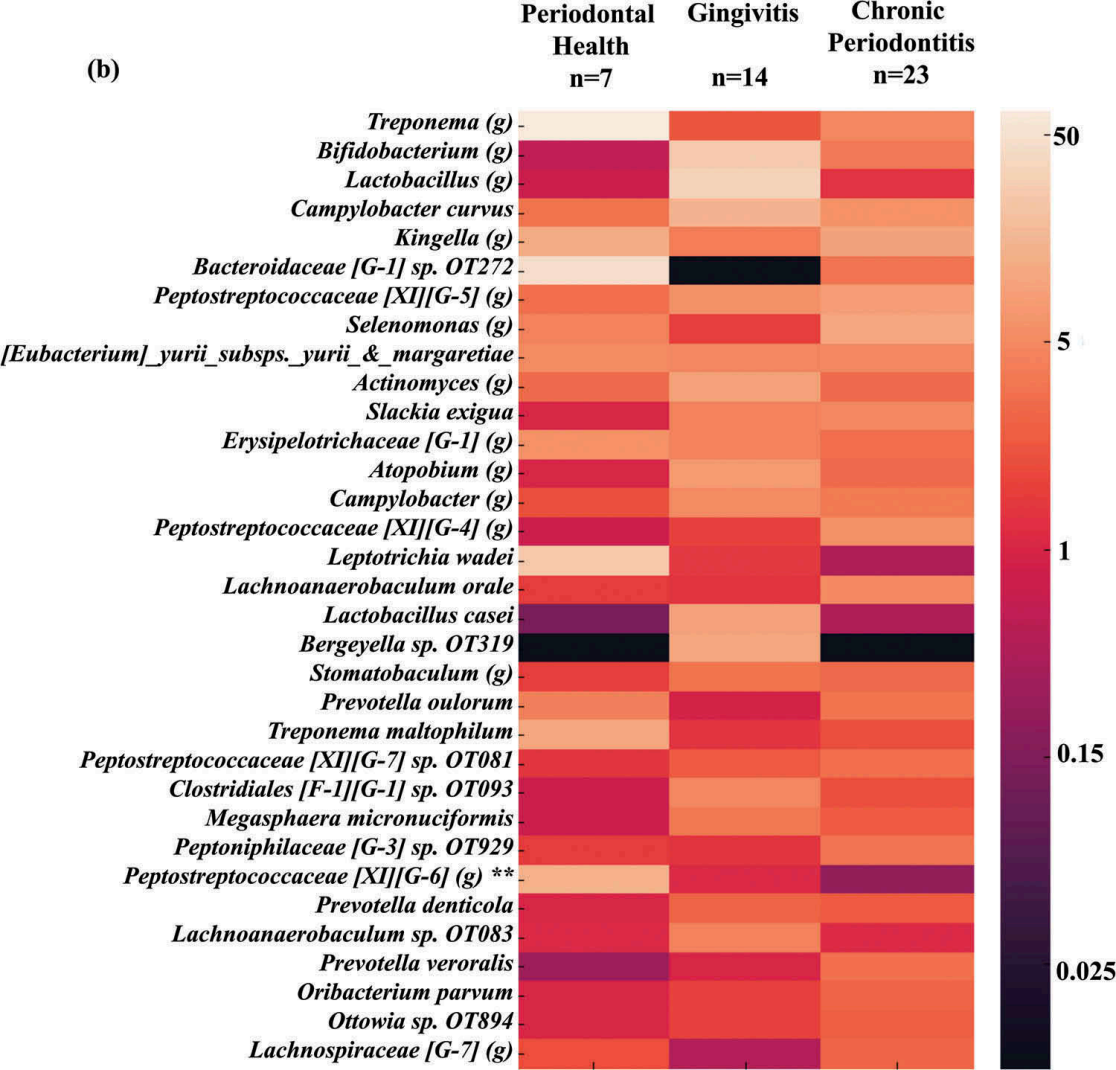
Continued.

## Discussion

In addition to bacteraemia and metastatic inflammation [17], another possible pathway associating periodontal diseases to systemic inflammatory diseases may be through alterations in the gut microbiome. In individuals with periodontal diseases, the long-term swallowing of high doses of periodontal pathogenic microorganisms could induce a dysbiosis of the intestinal microbiota, favouring the establishment of an ‘inflamed’ microbiome in terms of composition and/or function. In turn, this altered gut microbiota could modulate periodontal diseases by contributing to the progression and severity of periodontal tissue destruction. As a first approach to support this concept, we focused on determining the composition profile of the gut microbiome of individuals with periodontal diseases in comparison to periodontally healthy controls. We also searched for correlations between periodontal inflammation and attachment lost with specific microorganisms of the gut microbiota.

Along with our line of thought, some studies have addressed the possible association between periodontal disease/oral pathogens and the gut microbiota [45,46,52]. Arimatsu et al. [45] and Nakajima et al. [46] tested the direct impact of *P. gingivalis* on the gut microbiota through oral administration of high doses of this pathogen in mice. These authors reported an increase in local and systemic inflammation, glucose blood levels, insulin resistance, systemic endotoxemia, and a decrease in gut barrier function. Although no major changes in diversity were seen, significant changes in the gut microbiota composition of *P. gingivalis*-administered mice were observed, with an increase in *Bacteroidetes* (mainly the order *Bacteroidales*), a decrease in *Firmicutes*, and an increase in *Prevotella*. Of interest, the proportions of *Porphyromonadaceae* were still very low in these animals, suggesting that the pathobiont *P. gingivalis* may alter the gut microbiome not by outgrowing in the gut but by indirectly inducing endotoxemia. In fact, endotoxin-induced inflammation seems to be essential for the development of many metabolic disorders. For instance, Fei and Zhao [53] were able to induce obesity and insulin resistance in germ free mice inoculated with an endotoxin-producing bacterium (*Enterobacter cloacae* B29) isolated from a morbidly obese human’s gut.

In contrast to those previous studies in animal models [45,46], our findings showed that patients with periodontal diseases tend to present lower diversity in the gut microbiota. Other investigations have shown that reduced alpha diversity is a reliable indicator of disease-associated dysbiosis [1,54–56], corroborating our results. In relation to beta diversity, it was not possible to clearly distinguish individuals with distinct clinical status based on the composition of the gut microbiota.

Differences among clinical groups were observed in the composition of the gut microbiome at the phylum level. Considering the two major phyla commonly observed in the human gut microbiota, there was a tendency of *Bacteroidetes* to decrease and *Firmicutes* to increase with disease severity. In accordance with these data, a high *Firmicutes/Bacteroidetes* ratio in the gut microbiome has been reported in many other systemic inflammatory conditions [52,57]. Other investigations, however, have either reported a significant decrease in this ratio [45,46,58] or no changes at all [59] in systemic inflammatory conditions. Although the ratio *Firmicutes/Bacteroidetes* was high in our diseased population, these phyla varied widely in abundance among patients within the same clinical group (Figure S1). Other phyla increased in abundance in G and CP patients compared PH were *Proteobacteria, Verrucomicrobia* and *Euryarchaeota*. Multiple sclerosis patients have shown to present increased relative abundance of *Euryarchaeota* and *Verrucomicrobia* compared to healthy controls [60], whereas *Proteobacteria* is increased in severe acute malnutrition [61]. *Verrucomicrobia* is also increased in abundance in dextran sodium sulphate-induced murine colitis [62]. On the other hand, evidence indicates that the species *Akkermansia muciniphila* of the *Verrucomicrobia* phylum has a protective anti-inflammatory effect against specific metabolic disorders and obesity [63].

Variability in abundance of different microorganisms in stool samples was even greater at the genus level (Figure S2). Several genera related to putative periodontal pathogens such as *Fusobacterium, Prevotella, Parvimonas, Porphyromonas, Tannerella, Dialister, Filifactor, Treponema* and *Eubacterium* were detected in stool samples of all groups.

In particular, OTUs representative of *Prevotella, Comamonadaceae* (f), and *Lactobacillales* (o) were significantly abundant in patients with gingivitis, but the order *Bacteroidales* was increased in stools of PH patients. We also found significant positive associations between *Selenomonas noxia* and some genera of key periodontal pathogens (*Leptotrichia, Tannerella* and *Campylobacter*) in the gut with periodontal attachment loss. Finally, patients with periodontal diseases were accurately classified, and *Mogibacteriaceae* (f), *Ruminococcaceae* (f) and *Prevotella* (g) were the best predictors for classification of diseased patients. Contradictory results associating these gut microbial taxa with many different systemic diseases and health have been reported by others; therefore, results should be carefully interpreted. For instance, *Ruminococcaceae* has been related to a healthy gut [64], and *Prevotella* species are predominant commensal bacteria of healthy human mucosal sites [65]. Compelling data, however, have linked increased *Prevotella* abundance to inflammatory disorders, suggesting that some species exhibit distinct pathobiontic properties [65]. Inconsistency among studies may be partially due to the high inter-individual variability in gut microbiome composition at different taxonomic levels, as well as the complex multifactorial nature of periodontitis and systemic inflammatory diseases [66]. According to Duvallet and co-workers [54], there is not a unique dysbiotic microbiome associated with disease, and different diseases are characterized by distinct shifts in the gut microbiome. These shifts may be represented by depletion of beneficial species or enrichment of certain pathobionts. Of interest, many consortia of microorganisms in the gut microbiome are shared between a healthy and diseased state. This is true particularly at the genus level in which different strains or species within some genera play different roles in various microbiome-associated conditions. As an example, our data showed an association between increased abundance of *Prevotella* and periodontal disease (more specifically gingivitis). However, different species of this genus (*P. tannerae, Prevotella oulorum* and *Prevotella oris*) were detected in higher proportions in PH or in both PH and CP individuals. These authors have also reported that the order *Bacteroidales*, here abundant in periodontal health, was one of these organisms non-specifically associated with health or disease [54]. Additionally, confounding factors such as diet, smoking, obesity, and stress, among others have significant impact on the composition of the gut microbiome, and therefore on possible associations between specific consortia and disease. Of the life-style parameters here measured, only BMI was significantly higher in patients with periodontitis compared to other groups. Strong evidence has corroborated the positive association between obesity and periodontitis [67]. Therefore, the altered gut microbiome in overweight CP individuals may have contributed to the lower diversity and high numbers of pathogenic OTUs observed. Despite these caveats, OTUs representing periodontal pathogens were also detected in high numbers in PH controls and G who presented normal body weight. One limitation of the current study that may explain in part the lack of significant differences in microbial diversity among clinical groups or the existence of specific microbiome associations with health or disease is the small sample population evaluated, in particular in the control group. Challenges in patient compliance resulted in some stool submissions that were not in adequate conditions during the initial clinical examination and prior to dental treatment.

The frequent detection of oral species in stools samples of our study population was reinforced by comparing sequencing data against the HOMD 16S rRNA database [68]. OTUs representative of known pathogenic oral taxa including *P. endodontalis, C. rectus, D. invisus, P. micra, F. alocis, S. exigua, Treponema* spp., *Prevotella* spp., *Oribacterium* spp., *Tannerella* spp., *Leptotrichia* spp., *Selenomonas* spp. and *Fusobacterium* spp. were detected in high numbers in the gut microbiome of all patients regardless of periodontal status. Only a few species/phylotypes (*S. australis, Prevotella* spp., *R. aeria, G. adiacens, O. asaccharolyticum*, and *Porphyromonas* sp. OT930) were significantly more predominant in diseased individuals compared to PH. Many of these microorganisms are associated with caries, periodontal and endodontic infections [69–72], but they have also been isolated from extra-oral infections or systemic conditions [52–54,73–75]. In contrast, *S. australis* and *G. adiacens* have been related to periodontal health [69,70].

Koren et al. [56] brought up also the possibility of a link between oral and intestinal microbiota with inflammatory diseases by investigating whether the oral or gut microbiota could contribute to atherosclerosis. They reported that several OTUs from oral and gut sources were also detected in atherosclerotic plaque within the same patients. In addition to the shared OTUs between atherosclerotic plaques and oral/gut microbiota, some of these OTUs were strongly correlated with disease markers. For instance, *Fusobacterium* abundance in the oral cavity and members of the *Erysipelotrichaceae* and *Lachnospiraceae* families in the gut were positively correlated with LDL and total cholesterol. Taken all together, our results support the idea that a relatively large variety of oral species can gain access to the intestinal microbiota, regardless of the periodontal status. Whether these microorganisms colonize or not this body site, they may cause disturbances in this environment by releasing metabolites and cell components, which induce an inflammatory state [76]. They may also interact and enhance the pathogenic effects of other species that colonize the gut [77]. The mechanisms involved in the contribution of these oral species to intestinal dysbiosis are highly complex and beyond the scope of this study, but our data indicate that microorganisms of known pathogenicity and associated with periodontal and/or systemic inflammation were frequently detected in the gut microbiota of individuals with periodontal diseases.

In summary, the present data indicate that individuals with periodontal diseases present a less diverse intestinal microbiome, characterized by an increase in the *Firmicutes/Bacteroidetes* ratio, as well as an enrichment of the phyla *Euryarchaeota, Verrucomicrobia* and *Proteobacteria*, consistent with microbial shifts observed in some systemic inflammatory diseases. Moreover, high numbers of oral taxa related to periodontal destruction and inflammation were detected in the gut microbiome of these individuals regardless of periodontal status. Future approaches using animal models are required to investigate the direct or indirect possible mechanisms by which specific oral consortia may affect the composition and function of the gut microbiome. Furthermore, larger longitudinal clinical investigations are encouraged to determine the impact of periodontal treatment on the restoration of the gut microbiome, as well as the improvement of systemic health. A true causal relationship between periodontitis and gut dysbiosis will support the existence of an additional pathway linking periodontal diseases to other systemic inflammatory conditions, reinforcing the role of periodontal diseases as an important risk factor for these conditions.

## Supplementary Material

Supplemental Material
