# Supplementary material for: Defining the gut microbiota in individuals with periodontal diseases: an exploratory study
Source: J Oral Microbiol. 2018 Jul 3;10(1):1487741. doi: 10.1080/20002297.2018.1487741 (PMC6032013; doi:10.1080/20002297.2018.1487741)
Supplement: Supplemental Material [file ZJOM_A_1487741_SM8429.zip › supplemental files/Supplementary Information2.docx]

**Supplementary Information**

**Table S1.** 16S rRNA gene V4 variable region primers and final Illumina library amplification primers.

| **Primer name** | **Primer sequence (5’ -> 3’)** |
| --- | --- |
| PE16S_V4_U515_F* | ACACGACGCTCTTCCGATCTYRYRGTGCCAGCMGCCGCGGTAA |
| PE16S_V4_E786R* | CGGCATTCCTGCTGAACCGCTCTTCCGATCTGGACTACHVGGGTWTCTAAT |
| PE-III-PCR-F-### | AATGATACGGCGACCACCGAGATCTACACNNNNNNNNACACTCTTTCCCTACACGACGCTCTTCCGATCT |
| PE-IV-PCR-R-### | CAAGCAGAAGACGGCATACGAGATNNNNNNNNCGGTCTCGGCATTCCTGCTGAACCGCTCTTCCGATCT |

*Universal 16S rRNA gene underlined primer segments were adapted from (Lane, 1991).

**Table S2**. Demographic, clinical, behavioral and lifestyle characteristics of the study population.

|  | **PH**  **(n = 7)** | **G**  **(n = 14)** | **CP**  **(n = 23)** |
| --- | --- | --- | --- |
| Mean (SD) Age (years) * | 32.8 (15.7) | 28.4 (13.4) | 45.9 (11.6) |
| Gender (%)  Males  Females | 28.6  71.4 | 35.7  64.3 | 39.1  60.9 |
| Race (%)  White  African-American  Others | 71.4  0  28.6 | 78.6  0  21.4 | 60.9  17.4  21.7 |
| Smoking (%)  Non-smokers  Former smokers  Current smokers | 100  0  0 | 92.9  7.1  0 | 78.3  13.0  8.7 |
| Mean (SD) BMI in Kg/m^2^ * | 22.5 (2.9)^#^ | 23.6 (1.8)^#^ | 26.6 (2.7) |
| Physical Activity (%)  Sedentary  Light to moderate active  Very active | 42.9  14.3  42.9 | 62.5  37.5  0 | 55.0  25.0  20.0 |
| Alcohol consumption (%)  None  Light to Moderate  High | 50.0  16.7  33.3 | 66.7  0  33.3 | 64.7  5.9  29.4 |
| Mean (SD) n missing teeth * | 0.5 (0.8) | 1.4 (3.5) | 4.1 (2.8) |
| Mean (SD) PD * | 1.9 (0.3) ^#^ | 1.9 (0.1) ^#^ | 2.5 (0.5) |
| Mean (SD) CAL * | 1.9 (0.4) ^#^ | 1.9 (0.1) ^#^ | 2.7 (0.6) |
| *Mean (SD) % of sites with* |  |  |  |
| PD 5-6 mm * | 0^#^ | 0^#^ | 6.7 (6.8) |
| PD > 6 mm | 0 | 0 | 0.7 (1.7) |
| CAL 5-6 mm * | 0^#^ | 0.08 (0.2) ^#^ | 10.3 (9.3) |
| CAL > 6 mm * | 0^#^ | 0^#^ | 1.4 (2.5) |
| PL * | 25.7 (19.4) | 38.0 (14.2)^†^ | 48.7 (16.4)^†^ |
| GI * | 3.4 (5.5) | 22.2 (9.9)^†^ | 18.4 (12.9)^†^ |
| BOP * | 5.6 (3.4) | 16.5 (7.1)^†^ | 19.8 (11.5)^†^ |
| CA * | 6.0 (9.6)^#^ | 8.4 (6.9)^#^ | 22.4 (16.8) |
| SUP | 0 | 0 | 0.09(0.3) |

PH: Periodontal Health; G: Gingivitis; CP: Chronic Periodontitis; PD: probing depth; CAL: Clinical Attachment Level; PL: Visible Supragingival Plaque; GI: Marginal Gingival Bleeding; BOP: Bleeding on Probing; CA: Calculus; SUP: Suppuration; BMI: body mass index. * Significant differences among groups (Kruskal-Wallis test, p<0.01)^†^No significant differences between G and CP were observed; ^#^ No significant differences between PH and G were observed for these parameters (Mann-Whitney test, p>0.01).

**Table S3**. Mean (±SD) number of reads assigned to OTUs detected in feces samples from individuals with different periodontal status. OTUs detected at mean numbers >10 in all samples are presented.

| **OTUs** | **PH (n=7)** | | **G (n=14)** | | **CP (n=23)** | |
| --- | --- | --- | --- | --- | --- | --- |
| *Bacteroides (g)* | 7055 | ±11303 | 12428 | ±17789 | 4374 | ±6914 |
| *Clostridiales (o)* | 1258 | ±2096 | 6358 | ±19038 | 5737 | ±13270 |
| *RF39 (o)* | 507 | ±658 | 789 | ±1649 | 1183 | ±2281 |
| *Rikenellaceae (f)* | 652 | ±1106 | 1148 | ±1955 | 896 | ±2327 |
| *Fusobacterium (g)* | 2215 | ±5168 | 359 | ±707 | 739 | ±2445 |
| *Streptococcus (g)* | 353 | ±747 | 1954 | ±3180 | 214 | ±578 |
| *Bacteroidesuniformis* | 830 | ±1206 | 1199 | ±1726 | 298 | ±595 |
| *Ruminococcaceae (f)* | 460 | ±517 | 536 | ±686 | 636 | ±786 |
| *Lachnospiraceae (f)* | 404 | ±676 | 556 | ±587 | 453 | ±810 |
| *Parabacteroides (g)* | 333 | ±600 | 887 | ±1727 | 231 | ±337 |
| *Enterobacteriaceae (f)* | 21 | ±41 | 191 | ±420 | 474 | ±1851 |
| *Ruminococcus (g)* | 89 | ±115 | 330 | ±530 | 153 | ±310 |
| *Succinivibrio (g)* | 97 | ±183 | 57 | ±214 | 311 | ±1091 |
| *Faecalibacteriumprausnitzii* | 88 | ±156 | 244 | ±293 | 129 | ±157 |
| *Pseudomonas (g)* | 0 | | 487 | ±1263 | 1 | ±2 |
| *Prevotellacopri* | 74 | ±146 | 60 | ±111 | 210 | ±526 |
| *Christensenellaceae (f)* | 29 | ±64 | 23 | ±41 | 207 | ±655 |
| *Lachnospira (g)* | 153 | ±353 | 126 | ±184 | 79 | ±234 |
| *Neisseria subflava* | 16 | ±30 | 272 | ±890 | 30 | ±121 |
| *Odoribacter (g)* | 41 | ±65 | 193 | ±527 | 65 | ±133 |
| *Parvimonas (g)* | 179 | ±364 | 51 | ±91 | 103 | ±438 |
| *Coprococcuseutactus* | 34 | ±57 | 233 | ±605 | 21 | ±65 |
| *Prevotella (g) ** | 98 | ±206 | 157 | ±379 | 42 | ±180 |
| *Campylobacter (g)* | 154 | ±385 | 41 | ±97 | 79 | ±320 |
| *Prevotellamelaninogenica* | 89 | ±234 | 127 | ±288 | 43 | ±192 |
| *Coprococcus (g)* | 26 | ±59 | 56 | ±201 | 84 | ±311 |
| *Porphyromonas (g)* | 62 | ±127 | 145 | ±421 | 9 | ±34 |
| *Capnocytophaga (g)* | 105 | ±248 | 82 | ±219 | 33 | ±141 |
| *Paludibacter (g)* | 160 | ±420 | 12 | ±41 | 51 | ±240 |
| *Methanobrevibacter (g)* | 14 | ±25 | 68 | ±144 | 54 | ±91 |
| *Leptotrichia (g)* | 60 | ±102 | 37 | ±69 | 58 | ±253 |
| *Oscillospira (g)* | 25 | ±20 | 49 | ±78 | 49 | ±96 |
| *Leptotrichiaceae (f)* | 114 | ±301 | 63 | ±183 | 4 | ±19 |
| *Mogibacteriaceae (f)* | 49 | ±86 | 38 | ±50 | 38 | ±93 |
| *Tannerella (g)* | 81 | ±202 | 16 | ±40 | 40 | ±154 |
| *Prevotellananceiensis* | 21 | ±54 | 86 | ±223 | 11 | ±42 |
| *Proteus (g)* | 0 | | 113 | ±324 | 0 | |
| *Haemophilusparainfluenzae* | 24 | ±61 | 95 | ±312 | 2 | ±6 |
| *Dialister (g)* | 26 | ±34 | 36 | ±87 | 37 | ±75 |
| *Tissierella_Soehngenia (g)* | 0 | | 109 | ±281 | 0 | |
| *Porphyromonasendodontalis* | 119 | ±274 | 7 | ±17 | 22 | ±104 |
| *Gemellaceae (f)* | 47 | ±118 | 68 | ±121 | 5 | ±14 |
| *Bulleidia (g)* | 9 | ±16 | 71 | ±172 | 14 | ±49 |
| *Weeksellaceae (f)* | 13 | ±23 | 78 | ±199 | 5 | ±15 |
| *[Paraprevotellaceae] Prevotella (g)* | 7 | ±14 | 9 | ±16 | 46 | ±117 |
| *RF 32 (o)* | 3 | ±7 | 19 | ±64 | ±41 | 140 |
| *Veillonellaparvula* | 39 | ±76 | 40 | ±98 | 12 | ±37 |
| *Lactobacillales (o) ** | 6 | ±15 | 60 | ±97 | 9 | ±27 |
| *Dorea (g)* | 35 | ±84 | 17 | ±24 | 22 | ±32 |
| *Phascolarctobacterium (g)* | 6 | ±9 | 19 | ±28 | 28 | ±47 |
| *Providencia (g)* | 0 | 0 | 65 | ±188 | 0.04 | 0.2 |
| *Mogibacterium (g)* | 17 | ±29 | 38 | ±70 | 10 | ±24 |
| *Prevotellatannerae* | 105 | ±276 | 2 | ±5 | 6 | ±19 |
| *Filifactor (g)* | 21 | ±55 | 11 | ±30 | 23 | ±107 |
| *Erysipelotrichaceae (f)* | 30 | ±55 | 39 | ±70 | 2 | ±6 |
| *SR1 (p)* | 94 | ±247 | 8 | ±21 | 1 | ±4 |
| *Prevotellastercorea* | 0.4 | 0.8 | 0.4 | 1.2 | 33 | ±159 |
| *YS2 (o)* | 0 | | 18 | ±67 | 20 | ±92 |
| *Treponema (g)* | 67 | ±171 | 4 | ±10 | 7 | ±26 |
| *Rothiamucilaginosa* | 4 | ±11 | 31 | ±54 | 3 | ±10 |
| *Akkermansiamuciniphila* | 2 | ±4 | 14 | ±27 | 14 | ±26 |
| *Bacteroidales (o) ** | 38 | ±96 | 1 | ±3 | 9 | ±29 |
| *Blautiaproducta* | 0.14 | 0.4 | 0.14 | 0.5 | 21 | ±86 |
| *Clostridiaceae (f)* | 5 | ±8 | 9 | ±10 | 13 | ±25 |
| *Capnocytophagaochracea* | 15 | ±39 | 19 | ±67 | 4 | ±18 |
| *Bifidobacterium longum* | 1 | ±1 | 25 | ±63 | 5 | ±8 |
| *Comamonadaceae (f) * †* | 0 | | 20 | ±49 | 2 | ±5 |

OTU: operational taxonomic unit; PH: Periodontal Health; G: Gingivitis; CP: Chronic Periodontitis. (o): order taxonomic level; (f): family taxonomic level; (g): genus taxonomic level. Green cells refer to taxa detected in greater mean reads in the diseased groups in relation to healthy individuals. Orange cells refer to taxa detected in higher numbers in PH than diseased groups. Blue cells refer to taxa detected in high mean reads in both healthy and diseased individuals. * Refers to significant differences among groups (Kruskal-Wallis test, p<0.01). † Total mean number of reads = 7.

**Figure legends**

**Figure S1.** Relative abundance of different phyla in stool samples from individuals with different periodontal status Phyla detected at mean relative abundance ≥ 0.1% are presented.

**Figure S2.** Relative abundance of genera detected in stool samples from individuals with different periodontal status. Depicted are genera with > 0.1% mean relative abundance in all groups.

**Figure S3. Classification of periodontal disease based on OTUs detected in stool samples. (a)** An OTU count-based confusion matrix for patient classification. Columns represent the actual number of patients in each clinical condition and rows represent the number of patients predicted by OTU counts in each clinical group. Color represents the number of patient assignments, as indicated by the scale on the right. **(b)** Relative importance of contributing OTUs as determined by mean decrease in accuracy to classify periodontal health and disease.(f): family taxonomic level; (g): genus taxonomic level.

**Supplementary Reference**

Lane, D.J. (1991). 16S/23S rRNA sequencing. In Nucleic Acid Techniques in Bacterial Systemantics, E. Stackebrandt, and M. Goodfellow, eds. (Chinchester, England: Wiley & Sons), pp. 115–175.
