# Supplementary figures and images for: Defining the gut microbiota in individuals with periodontal diseases: an exploratory study
Source: J Oral Microbiol. 2018 Jul 3;10(1):1487741. doi: 10.1080/20002297.2018.1487741 (PMC6032013; doi:10.1080/20002297.2018.1487741)

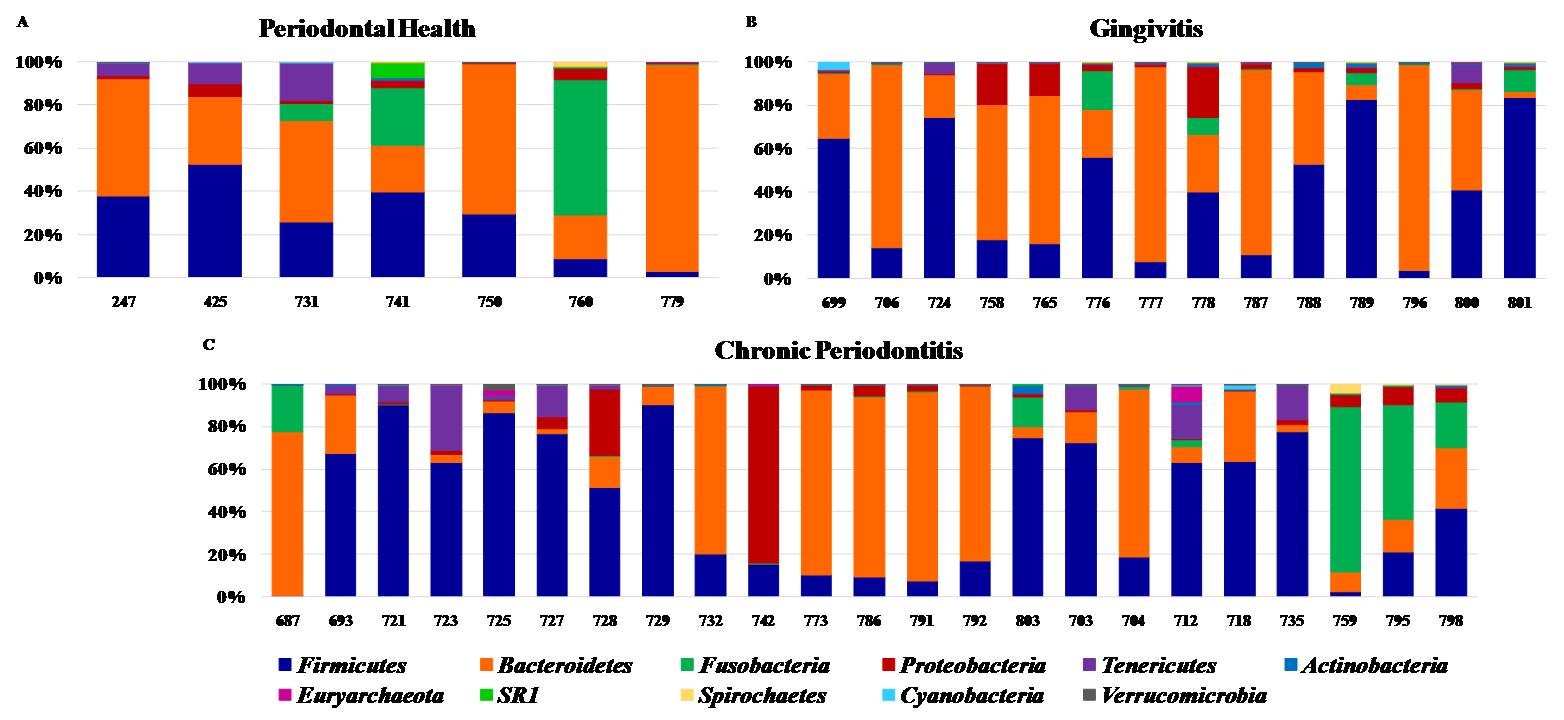

Supplement: Supplemental Material [file ZJOM_A_1487741_SM8429.zip › supplemental files/Figure S1.jpg]

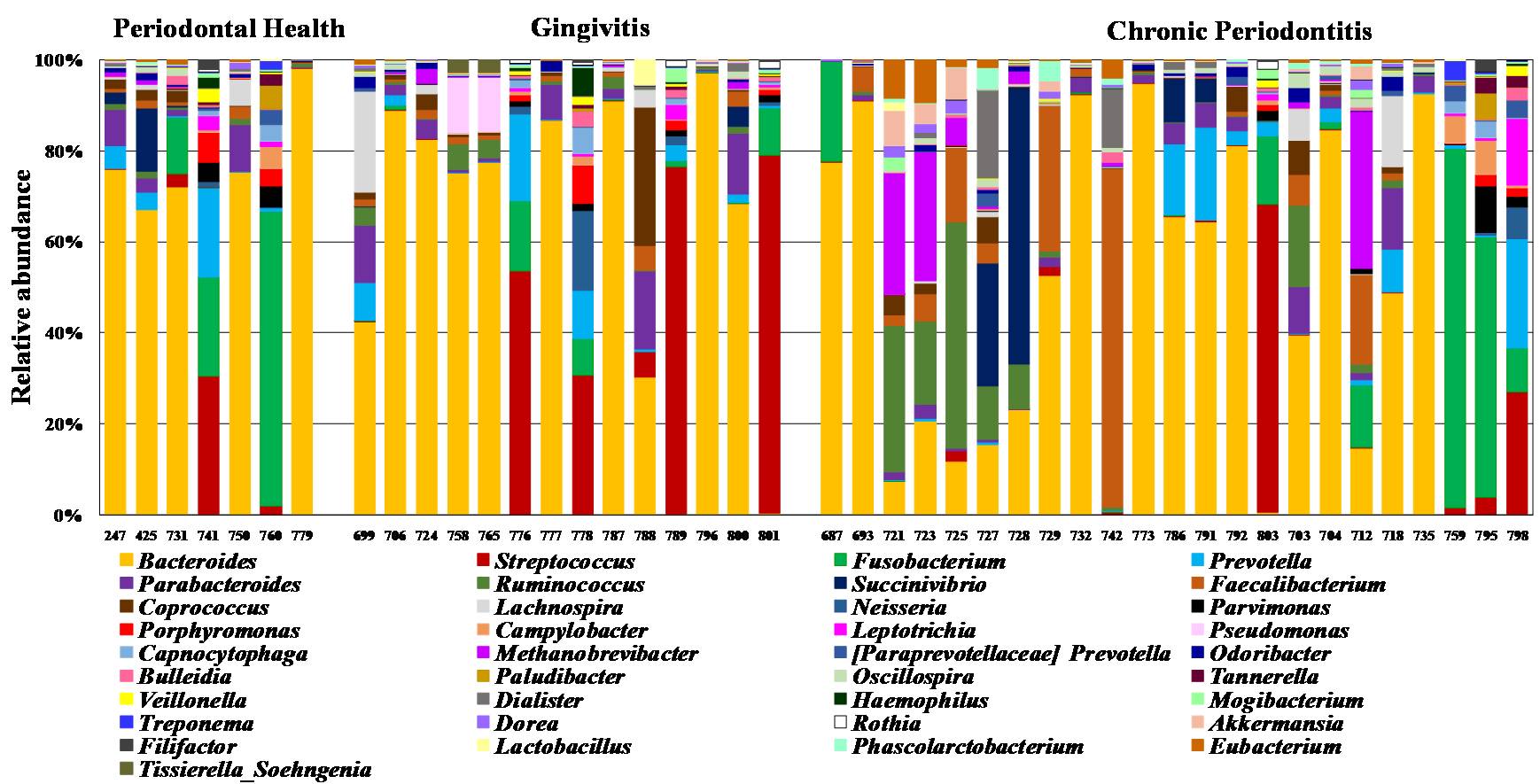

Supplement: Supplemental Material [file ZJOM_A_1487741_SM8429.zip › supplemental files/Figure S2.jpg]

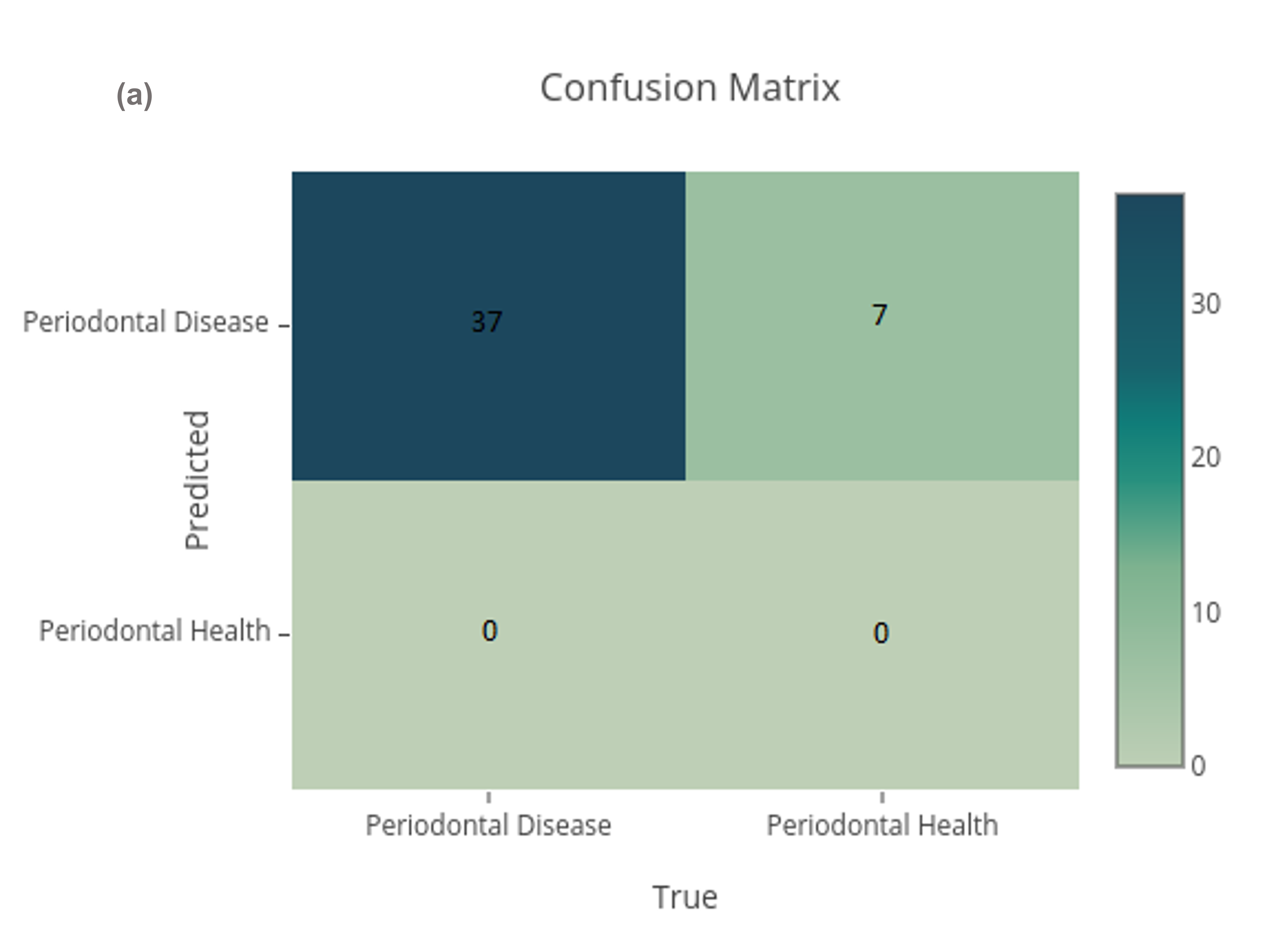

Supplement: Supplemental Material [file ZJOM_A_1487741_SM8429.zip › supplemental files/Figure S3a.png]

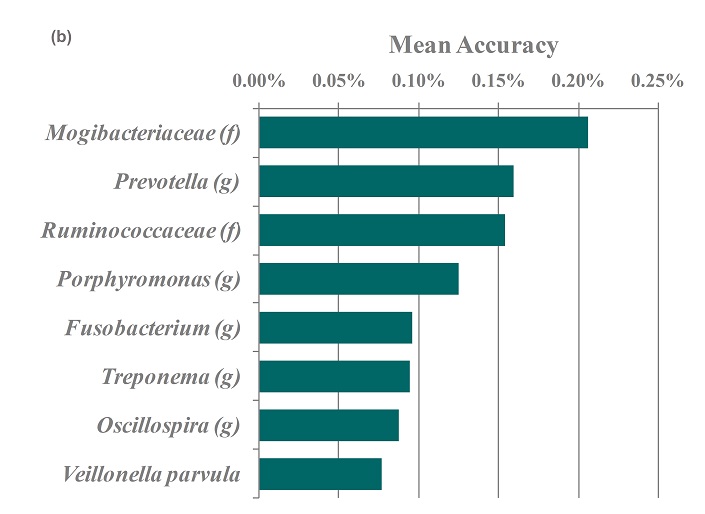

Supplement: Supplemental Material [file ZJOM_A_1487741_SM8429.zip › supplemental files/Figure S3b.jpg]
